# *Actinomyces meyeri*: A Rare Cause of Postsurgical Pelvic Actinomycosis

**DOI:** 10.1155/2018/3842048

**Published:** 2018-06-21

**Authors:** C. Michele Markey, Lauren E. Vestal

**Affiliations:** University of Missouri-Kansas City School of Medicine, Kansas City, MO, USA

## Abstract

*Actinomyces meyeri* bacterium resides on mucosal surfaces and is uncommonly pathogenic. When* A. meyeri *does cause infection, these infections are typically pulmonary in origin and have the capacity to disseminate throughout the body.* A. meyeri* is an uncommon cause of pelvic infection. We present a unique case of a posthysterectomy abscess caused by this particular bacterium.

## 1. Introduction

*Actinomyces* is a bacterium that resides commensally on mucosal surfaces of the oral, gastrointestinal, and genital tracts. It uncommonly causes infection; however, when it does, the infection is typically chronic and granulomatous in nature. Infection is more common in men and tends to be an indolent disease [1].

Primary abdominopelvic actinomycosis typically mimics an intra-abdominal malignancy due to its potential to form an infiltrative mass, whereas postoperative actinomycosis presents more often as an abscess. The most common species responsible for infections is* Actinomyces israelii* [2]. We present a case of postoperative pelvic actinomycosis as the result of* Actinomyces meyeri*, which is, to our knowledge, the first reported case of such an infection due to this species of bacteria.

## 2. Case Report

A 40-year-old gravida five, para four woman presented for workup and management of abnormal uterine bleeding. Her past medical history was significant only for hypertension and anemia. On review of her social history, she admitted to drinking six packs of beer on the weekends but denied further substance use. She denied previous treatments for her bleeding including any previous intrauterine device usage.

Ultrasonography revealed a 7 cm fundal fibroid with otherwise normal pelvic anatomy. She was initially offered medical management of her bleeding. She declined any medical treatment and strongly desired definitive surgical treatment. She then underwent a total vaginal hysterectomy with adnexal conservation. Due to the large size of the uterus, a myomectomy was performed to facilitate vaginal removal. Her postoperative hospital course was relatively uncomplicated and she was discharged home on postoperative day three.

On postoperative day ten, she presented to the Emergency Department (ED) for fever, worsening abdominal pain, and new onset of nausea and vomiting. In the Emergency Department, she was tachycardic and tachypneic but afebrile. Her exam was significant for abdominal tenderness to minimal palpation, vaginal cuff erythema, and significant tenderness to palpation of the vaginal cuff. Lab work showed an elevated white blood cell count. She was admitted for management of presumed pelvic infection.

**Figure 1:**
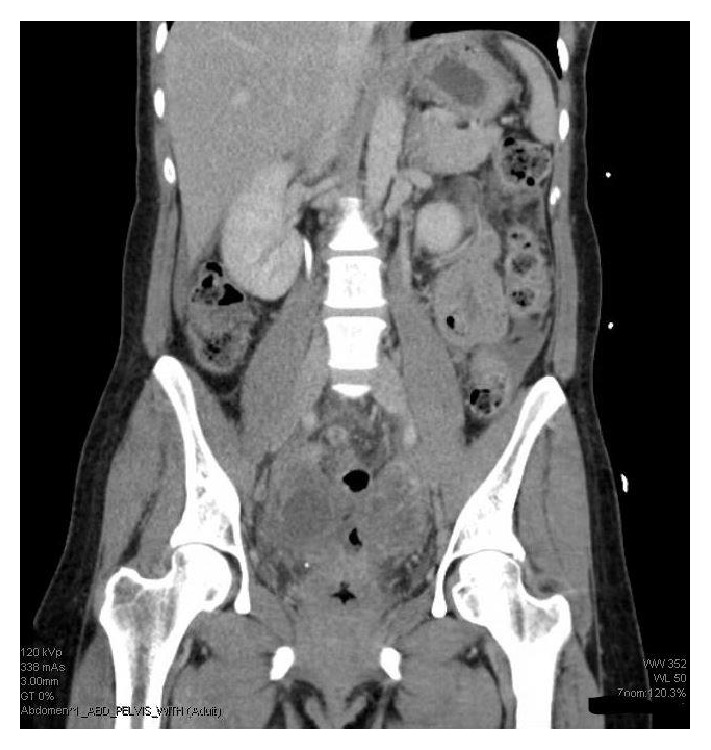
Coronal CT image demonstrating abscess at vaginal cuff.

**Figure 2:**
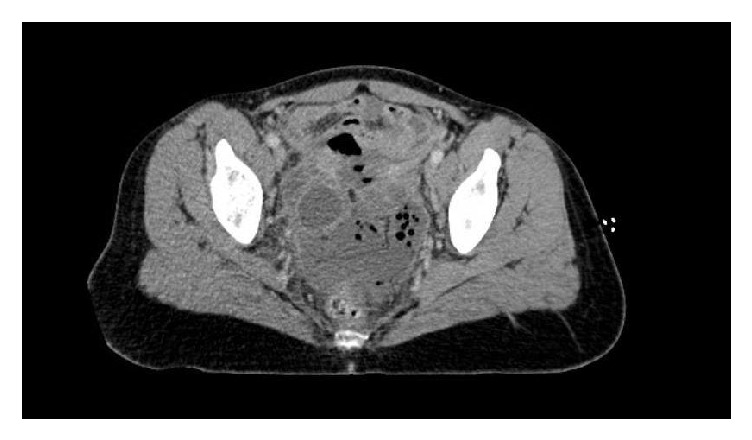
Axial CT image demonstrating abscess at vaginal cuff.

A CT of the abdomen and pelvis was obtained and showed a 6.2 x 9.7 cm pelvic abscess adjacent to the vaginal cuff (Figures 1 and 2).

Interventional Radiology placed a drain into the abscess and the patient was started on IV piperacillin/tazobactam. She was transitioned to oral amoxicillin/clavulanate potassium after four days on intravenous antibiotics and her drain was removed on hospital day 5. Vaginal wound cultures remained pending; however, due to continued clinical improvement on the oral antibiotic regimen, she was discharged home on hospital day 5 with a two-week course of amoxicillin/clavulanate potassium.

The patient then returned for her outpatient visit approximately one week later. The results of the vaginal wound cultures revealed a large growth of* Actinomyces meyeri*. The patient's case was discussed with an Infectious Disease (ID) specialist who recommended an additional two-week course of amoxicillin/clavulanate potassium.

The patient then returned to the ED on postoperative day 25 for pleuritic chest pain with mild cough but denied gynecologic complaints. She reported compliance with the oral amoxicillin/clavulanate potassium regimen. Exam and lab work were unremarkable. A chest X-ray showed left basilar heterogeneous opacities, likely subsegmental atelectasis. A CT angiogram was obtained due to concern for a possible pulmonary embolism (PE). The imaging was negative for a PE; however, it was concerning for possible pneumonia. The patient was discharged home with a five-day course of levofloxacin for treatment of pneumonia.

On postoperative day 27, the patient represented to the ED with worsening shortness of air and chest pain. Again, she reported compliance with her antibiotic prescriptions. Exam and lab work were again unremarkable. A repeat chest X-ray showed a slight progression of right basilar heterogeneous opacities thought to be infectious. Her antibiotic regimen was again discussed with ID specialists and an intravenous antibiotic regimen was felt preferable to an oral antibiotic course. She then completed an outpatient two-week course of IV ampicillin/sulbactam as recommended.

On postoperative day 37, a repeat CT of the abdomen and pelvis showed near complete resolution of the previous pelvic abscess. HIV testing was obtained and returned negative result. She reported significant improvement of her symptoms. She was placed on a six-month course of oral amoxicillin per ID recommendations with plans for continued follow-up in their clinic, as well as with gynecology. She has not shown any signs of recurrent infection after approximately 1 year of follow-up.

## 3. Discussion

*Actinomyces* is normally present commensally on mucosal surfaces of the oral, gastrointestinal, and genital tracts; however, it may gain access to tissues and cause infection through any insult that disrupts the mucosal barrier, such as surgical procedures or trauma. When infection is caused by this bacterium, the infection is typically chronic and granulomatous in nature and is most often caused by* Actinomyces israelii* [1, 2]. Actinomycosis is more common in men and tends to be indolent. Primary abdominopelvic actinomycosis typically mimics an intra-abdominal malignancy due to its potential to form an infiltrative mass, whereas postoperative actinomycosis presents more often as an abscess [3]. Most commonly, pelvic actinomycosis in females has been linked to prolonged intrauterine device usage. The common strain isolated in these cases is* A. israelii*; however,* A. turicensis, A. naeslundii, A. odontolyticus, *and* A. gerencseriae* have also been isolated. Characteristically, sulfur granules are found on microscopic examination of pus samples in cases of actinomycosis. [1] Treatment is a long duration of antimicrobial therapy with treatment lengths lasting up to 1 year in some cases.

*Actinomyces meyeri* is an uncommon cause of actinomycosis [3]; however, there is some evidence that it may be more common than previously recognized [4].* A. meyeri* was first isolated from a lung empyema in 1911 [5]. The oropharynx is currently thought to be the normal niche of* A. meyeri* [6]. Most commonly,* A. meyeri* is associated with pulmonary infections thought to be secondary to aspiration [3, 7]. One unusual feature of pneumonia caused by* A. meyeri* is systemic dissemination, most frequently to skin, long bones, liver, brain, and muscles [3]. Risk factors for infection with* A. meyeri* are alcoholism and periodontal disease [3].

*A. meyeri* has also been found to be an uncommon source of obstetrical infection [8]. These cases are described in a recent literature review published in 2017, which found 17 cases of obstetric related* Actinomyces* infections. A particular species of* Actinomyces* was identified in only five of these cases, with the identified species including* A. israelii*,* A. neuii*, and* A. meyeri* [8]. Additionally, a single report describes a case of fatal sepsis related to pyomyoma; however, this infection was not following any surgical intervention or instrumentation of the uterus. Blood cultures were polymicrobial and included* A. meyeri* growth; however, cervical and uterine abscess cultures grew only* Staphylococcus aureus* [9].

This case report reviews a postsurgical pelvic actinomycosis caused by* A. meyeri.* Postoperative pelvic infection caused by* A. meyeri* has yet to be described in the literature. An interesting aspect in this case is the patient's subsequent development of pneumonia. Given the previously described ability for* A. meyeri* infections to disseminate, especially when they are pulmonary in origin, one may wonder if the original infection was genital or pulmonary in origin. The infection may have potentially begun insidiously in the lungs and disseminated to the pelvis. The surgical procedure then may have provided a nidus of tissue damage from which the infection could thrive and develop into an abscess. However, the timeline of this case strongly suggests that the infection was genital in origin. Potentially, the infection may have disseminated from the pelvis to the lungs. Unfortunately, no sputum cultures were obtained and, as such, the pathologic organism of the pneumonia is unknown and certainly cannot be assumed to be* Actinomyces meyeri*.

Given that the treatment of actinomycosis is long-term antibiotics, with regimens sometimes lasting up to a year, the impact of these types of infections is much larger than postoperative abscesses caused by more typical agents. As such, more research is needed to not only identify the causative actinomycosis species but also establish means of early detection or prevention of these infections.

## 4. Conclusion

*Actinomyces meyeri* is a previously unknown cause of postoperative pelvic infections. In our patient,* A. meyeri* caused a postoperative abscess that required treatment with two weeks of intravenous antibiotics followed by six months of oral antibiotic therapy. Due to the extensive antibiotic treatment required to resolve infections from this organism, prevention and early detection of these infections are paramount.
